# Spina Bifida Incidence Trends: A Comparative Study of Puerto Rico and the United States

**DOI:** 10.3390/epidemiologia6040092

**Published:** 2025-12-16

**Authors:** Eric Pérez-Pérez, Esteban Rivera-Rivera, Natasha Frontera, Alejandro Cedeño-Moran, Camelia Carvajal-Matta, Jeremy González, Aixa de Jesús-Espinosa, Iván Sosa-González, Miguel Mayol del Valle

**Affiliations:** 1 School of Medicine, University of Puerto Rico, San Juan 00931, Puerto Rico; eric.perez5@upr.edu (E.P.-P.); alejandro.cedeno@upr.edu (A.C.-M.); camelia.carvajal1@upr.edu (C.C.-M.); jeremy.gonzalez1@upr.edu (J.G.); 2 Section of Neurosurgery, Department of Surgery, School of Medicine, University of Puerto Rico, San Juan 00931, Puerto Rico; esteban.rivera6@upr.edu (E.R.-R.); aixa.dejesus@upr.edu (A.d.J.-E.); ivan.sosa@upr.edu (I.S.-G.); 3 Department of Surgery, School of Medicine, University of Puerto Rico, San Juan 00931, Puerto Rico; natasha.frontera@upr.edu

**Keywords:** neural tube defects, myelomeningocele, spina bifida, folic acid, congenital defects

## Abstract

Objectives: Neural tube defects such as myelomeningocele (MMC) remain a significant public health concern despite prevention efforts. Public health measures have reduced the global MMC incidence, but socioeconomic disparities may limit their impact. Puerto Rico (PR) is a United States (US) territory; however, its socioeconomic landscape is vastly different, which may contribute to differences in MMC incidence. In this study, we aimed to compare the differences in MMC incidence and annual variability between PR and the US. Materials and Methods: Data on MMC incidence for the US was obtained from the Centers for Disease Control’s National Vital Statistics Reports, and data for PR from the Puerto Rico Birth Defects Surveillance and Prevention System. Annual percentage change (APC) was used to evaluate year-to-year variation, and multiple linear regression analysis was applied to compare incidence rates. Results: The mean annual MMC incidence in 1996–2020 was 4.88 per 10,000 live births in PR (SD = 1.86), and 1.78 (SD = 0.35) in the US, with an estimated mean difference of 3.11 (*p* < 0.001). APCs during this period varied significantly, ranging from +200% to −63%. A subgroup analysis after folic acid fortification efforts in PR (1999–2020) showed a persistently elevated incidence in PR (mean = 4.41, SD = 1.33) vs. US (mean = 1.67, SD = 0.25), with an estimated mean difference of 2.72 (*p* < 0.001). Conclusions: Despite folic acid fortification and public health interventions, MMC incidence in PR remains higher and more variable. These findings underscore the need for improved disease reporting and targeted, region-specific preventive strategies.

## 1. Introduction

Spina bifida is a congenital neural tube defect (NTD) resulting from incomplete closure of the embryonic neural tube during the fourth week of gestation. It includes a spectrum of anomalies, classified into spina bifida occulta, a closed form typically covered by skin, and spina bifida aperta, which encompasses meningocele, myelomeningocele (MMC), and myeloschisis [1,2]. Among these, MMC represents the most severe and clinically significant subtype, characterized by the herniation of meninges and neural elements through a vertebral defect [2]. The exposed neural tissue undergoes progressive intrauterine injury, resulting in neurological impairment that varies according to the level of the lesion [1].

Myelomeningocele (MMC) occurs in approximately 1 in 1000 births worldwide, making it one of the most common congenital malformations [2]. In the US, historical surveillance data estimate the overall incidence of spina bifida at approximately 3 per 10,000 live births, with MMC accounting for the majority of these cases [2]. The condition is frequently associated with significant comorbidities, including Chiari II malformation and hydrocephalus in over 80% of cases [3,4,5]. Orthopedic complications such as scoliosis, as well as urological issues including neurogenic bladder and chronic kidney disease, are also common [3,6,7]. Delays in surgical intervention further increase the risk of cerebrospinal fluid leakage, pseudomeningocele formation, and intracranial infection [3,8]. As a result, lifelong multidisciplinary management is essential to address the wide-ranging medical, surgical, and psychosocial needs of affected individuals. Beyond the clinical implications, caring for children with MMC also imposes a considerable emotional, psychological, and economic burden on caregivers, who often experience elevated anxiety, depressive symptoms, and reduced quality of life [9].

The clinical burden of MMC is significant, and understanding its underlying causes is crucial for prevention efforts. Genetic predisposition is a recognized risk factor for NTDs, with several chromosomal abnormalities linked to increased risk, although no single gene has been definitively implicated [10,11]. Additional risk factors, such as maternal obesity and exposure to environmental toxins, have also been involved in increasing the risk of NTDs [12]. Most cases of NTDs result from disorders of the folate pathway and are largely preventable with folate supplementation [13]. This is of particular significance in teenage pregnancies, which often lack prenatal care, resulting in inadequate folic acid intake and increased risk of NTDs [14]. However, approximately 30% of myelomeningocele cases are estimated to occur independently of folic acid deficiency, highlighting the contribution of non-folate-related factors [15,16].

Although efforts to reduce the incidence of myelomeningocele have advanced significantly, regional disparities remain. In high-income countries, such as Canada and the United States (US), public health efforts, including the widespread implementation of folic acid supplementation and improved prenatal care, have contributed to a decline in spina bifida incidence [17]. Despite these interventions, socioeconomic factors and unequal access to healthcare services have persisted [13]. Many low- and middle-income countries continue to report higher rates of myelomeningocele, often attributable to poor maternal nutrition, inadequate prenatal care, and limited access to preventive services [12]. Understanding these disparities is crucial, particularly in regions like Puerto Rico (PR), where genetic, environmental, and healthcare access factors intersect in unique ways. This study aims to explore and compare trends in myelomeningocele incidence between PR and the mainland US to inform future better-targeted prevention strategies. A comparison was conducted between the US and PR, given PR’s status as a US territory, which theoretically grants access to the same federal preventive health initiatives. This direct comparison within a shared policy and regulatory framework enables a more precise assessment of implementation disparities and region-specific challenges, rather than focusing solely on socioeconomic differences. By analyzing trends in incidence and temporal variability, this comparison can help identify gaps in existing public health initiatives, justify resource allocation, and inform the development of culturally and regionally tailored prevention and education programs.

## 2. Materials and Methods

We conducted a retrospective descriptive epidemiological study analyzing the incidence and trends of myelomeningocele from 1996 to 2020. Data for PR were obtained from the Puerto Rico Birth Defects Surveillance and Prevention System annual reports [18]. US data were retrieved from the CDC National Vital Statistics Reports. Cases were included as reported by each source and were not reclassified by diagnostic codes. Only live births were included.

Incidence rates of MMC per 10,000 live births were extracted annually. We calculated annual percentage change (APC) to assess year-to-year variations and applied a multiple linear regression model to estimate differences in crude incidence rates between PR and US, adjusting for year. The regression model did not adjust for confounders due to limitations in the dataset. To explore temporal consistency and mitigate the influence of early data variability, we conducted a sub-analysis focusing on 1999 through 2020. This decision was informed by notably elevated and erratic incidence values observed in PR from 1996 to 1998, which may have reflected transitional inconsistencies in surveillance or delayed implementation of public health measures. As in the primary analysis, incidence rates per 10,000 live births for PR were obtained from the previously mentioned annual reports. We calculated descriptive statistics, including the mean, standard deviation (SD), and range for each region, and fitted regression models with 95% confidence intervals (CIs) to compare trends between PR and the US. The analysis was performed using R version 4.2.2.

This epidemiological analysis was complemented by a literature review exploring possible explanatory variables behind the observed trends, including environmental exposures, folic acid intake, metabolic conditions, genetic factors, and reproductive health education.

## 3. Results

### 3.1. Myelomeningocele Incidence

**Figure 1:**
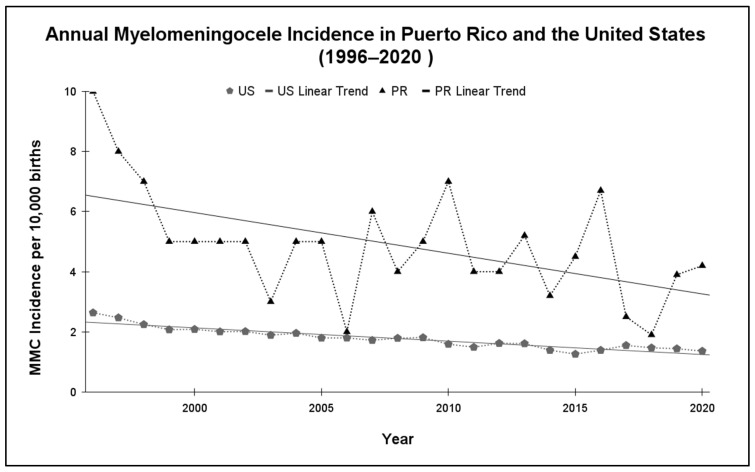
Annual MMC prevalence per 10,000 live births in PR and the US, 1996–2020. The US displays a strong declining linear trend. PR’s trendline is more variable, indicating instability in long-term progress. Abbreviations: Myelomeningocele (MMC), United States (US), and Puerto Rico (PR).

Between 1996 and 2020, MMC incidence in PR ranged from 10.0 to 1.9 cases per 10,000 live births, consistently higher than US rates (2.64 to 1.26 per 10,000 live births) (Figure 1). Descriptive analysis showed that the mean annual incidence of MMC in PR was 4.88 cases per 10,000 live births (SD = 1.86), while the mainland US had a mean of 1.78 cases per 10,000 live births (SD = 0.35). Over this period, MMC incidence in PR was significantly higher than in the mainland US, with an estimated average difference of 3.11 cases per 10,000 live births (95% CI: 2.42–3.77; *p* < 0.001) after controlling for year-to-year variation.

### 3.2. Myelomeningocele Annual Variability

**Figure 2:**
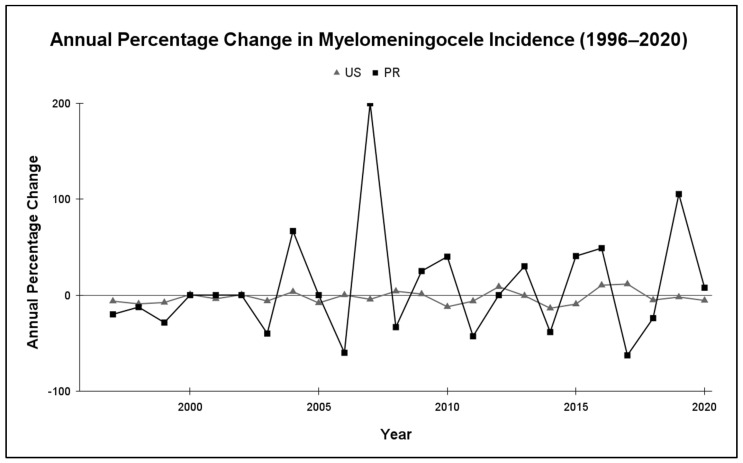
Annual percentage change (APC) in MMC incidence in PR and the US, 1996–2020. PR’s APC shows large year-to-year fluctuations, while the US data demonstrate minimal variation and remain relatively stable around zero. Abbreviations: United States (US) and Puerto Rico (PR).

Annual percentage change (APC) analysis (Figure 2) revealed substantial year-to-year variability in PR, ranging from +200% to −63%. In contrast, the US showed a gradual and stable decline in incidence. PR exhibited nearly 9 times the year-to-year variability as the US.

### 3.3. 1999–2020 Subgroup Analysis

Between 1999 and 2020, the mean incidence of MMC in PR was 4.41 cases per 10,000 live births (SD = 1.33; range: 1.9–7.0), while the mainland US reported a mean of 1.67 cases per 10,000 live births (SD = 0.25; range: 1.26–2.09). These values reflect a more consistent reporting period and reduce the skewing effect of the earliest years in the dataset. During this sub-period, the MMC incidence in PR remained significantly higher than that of the mainland US, with an estimated average difference of 2.72 cases per 10,000 live births (95% CI: 2.15–3.30, *p* < 0.001). This sub-period had a narrower SD than the whole dataset, particularly in PR, suggesting a reduction in year-to-year variability but not a resolution of the baseline disparity.

## 4. Discussion

The results of this study highlight notable differences in the epidemiological behavior of myelomeningocele incidence between PR and the mainland US over the 25-year study period. PR exhibited a consistently higher incidence of MMC, with a mean of 3.96 cases per 10,000 live births compared to 1.83 in the US. As shown in Figure 1, the US followed a gradual, stable downward trend in MMC prevalence. In contrast, PR’s trendline showed limited long-term improvement and considerable year-to-year variation. This variability is further underscored by the annual percentage change analysis in Figure 2, which revealed marked fluctuations in PR’s APC, with abrupt increases and decreases in incidence that were not observed in the US.

A sub-analysis was performed to better understand the consistency of myelomeningocele incidence over recent decades, excluding data from 1996 to 1998, a period marked by elevated, highly variable rates in PR. In the US, mandatory fortification of enriched cereal grain products with folic acid was authorized in 1996 and implemented over the following years [19]. The early fluctuations in our results may reflect inconsistencies in reporting or a lag in the implementation of folic acid fortification measures in PR compared to the mainland US. We performed a sub-analysis of MMC incidence from 1999 through 2020 to mitigate this variability. In this 1999–2020 sub-analysis, PR maintained a mean MMC incidence of 4.41 cases per 10,000 live births (SD = 1.31; range: 1.9–7.0), while the US had a lower, more stable mean of 1.68 (SD = 0.25; range: 1.26–2.09). The resulting incidence rate ratio increased to approximately 2.61, indicating that the disparity persisted even after excluding the earliest years. These patterns highlight ongoing differences in temporal variability and sustained incidence between the two regions, warranting further examination of contextual factors that may contribute to this divergence.

Although based on surveillance data, this study advances our current knowledge by quantifying annual variability and long-term trends in MMC incidence within and between PR and the mainland US. These temporal patterns reveal persistent disparities that warrant further investigation into underlying determinants and guide the design of future analytical studies and targeted prevention efforts. In the following discussion, we describe potential differences in region-specific factors that may influence reporting patterns, prevention efforts, and risk exposure over time. These include genetic susceptibility, preconception health behaviors, metabolic factors, disparities in folic acid intake, inconsistencies in reproductive health education, and differences in public health interventions.

### 4.1. Genetic Susceptibility: MTHFR C6677T Variant and NTDs

NTDs, including MMC, have been linked to genetic variants in folate metabolism. The incorrect neural tube closure during embryogenesis, which is impacted by genetic and environmental factors, is the cause of NTDs, including MMC. For example, mutations in the 5,10-methylenetetrahydrofolate reductase (MTHFR) gene, notably the C677T variant, result in reduced enzyme activity, leading to elevated homocysteine levels and depletion of 5-methyltetrahydrofolate, thereby disrupting the folate-dependent processes critical for neural tube closure [20]. García-Fragoso et al. found that in PR, 19% of mothers with children affected by NTDs displayed homozygosity for this mutation, compared to 9% in the control group [21]. In the mainland US, the prevalence of C677T mutation homozygosity varies, but has been reported to be approximately 10–15% amongst white North Americans [22]. Although these results show varying frequencies of the C677T mutation across populations, further investigation is needed to determine if these variations significantly contribute to the disparities in NTD incidence observed between PR and the US.

### 4.2. Maternal Substance Use: Alcohol and Tobacco

Maternal substance use, particularly tobacco and alcohol consumption during pregnancy, has been identified as a modifiable risk factor for NTDs, including MMC. Exposure to these substances during pregnancy may increase the risk of NTDs by causing oxidative stress and metabolic disruptions that hinder the folate-dependent cell proliferation required for appropriate neural tube closure [23,24]. Data from the USA Pregnancy Risk Assessment Monitoring System (PRAMS), which analyzed the self-reported behavior of 36,493 women in 37 US jurisdictions, shows that, in 2021, the prevalence of cigarette smoking during pregnancy in PR was 0.4% (*n* = 965), while in the continental US, the prevalence was 5.9% (*n* = 35,528) [25]. Although further research is needed, this data suggests that cigarette use during pregnancy is not responsible for the observed difference in prevalence of NTDs in PR. With respect to alcohol use, the CDC analyzed 2018–2020 Behavioral Risk Factor Surveillance System (BRFSS) self-reported data from 6327 pregnant adults aged 18–49 years from all 50 US states and the District of Columbia, and found that 13.5% reported current drinking (at least one alcoholic drink in the past 30 days) and 5.2% reported binge drinking while pregnant [26]. A study on substance use disorders and treatment needs conducted in 2008 surveyed 3180 individuals in PR between the ages of 15 and 74 using a self-administered questionnaire and found that the rate of alcohol use among women who had been pregnant during the past 12 months was 47.1%. They also found that one in seven (13.6%) women who had been pregnant during the past 12 months met criteria for alcohol abuse, and one in eighteen (5.5%) met criteria for alcohol dependence [27]. However, a more recent report in 2023, published by the Puerto Rico Department of Health, found that 2–4% of pregnant women consume alcohol [28]. However, this report only evaluates alcohol use during the last three months of pregnancy. Even though PR has lower rates of smoking compared to the continental US, a potentially higher rate of alcohol consumption in PR compared to the continental US during the first months of pregnancy could explain the differences in MMC rates. While significant differences in maternal substance use between PR and US populations have been identified, further studies are needed to clarify the exact relationship between these differences and their role in the existing MMC disparities between US and PR populations.

### 4.3. Maternal Metabolic Factors: Obesity, Diabetes, and Risk of MMC

Maternal factors such as obesity and diabetes mellitus (DM) increase the risk of significant congenital abnormalities, especially those of the nervous system [29]. In these conditions, chronic inflammation and insulin resistance synergistically contribute to NTDs by promoting oxidative stress, dysregulating developmental genes, and inducing apoptosis in the neuroepithelium [30,31]. These disruptions, driven by pro-inflammatory cytokines, impaired autophagy, and metabolic stress, interfere with proper neural tube closure during embryogenesis. While current guidelines suggest that all women of reproductive age should consume 400 μg of folic acid per day, they also acknowledge that women who are more susceptible to NTDs, such as those who are obese, may need higher doses. Still, no specific adjustment is recommended [32].

Currently, no US study has directly examined the effects of prescribing higher folic acid dosages to pregnant women who are obese, nor whether increased supplementation improves neurodevelopmental outcomes in this population [32]. Therefore, more research is necessary to ascertain whether the folic acid recommendations currently in place are adequate for obese women and whether customized dosing strategies could more effectively address their unique risk profile.

The incidence of NTDs in pregnancies of mothers without diabetes is substantially lower compared to women experiencing gestational diabetes mellitus (GDM) or pre-pregnancy DM. The baseline prevalence of NTDs in the general population in North America is approximately 0.03–0.09% (34–87 per 100,000 live births), with folic acid supplementation and food fortification programs having reduced rates further [13,33]. The risk ratio for MMC was 2.0 in individuals with pre-pregnancy DM and 1.13 in those with GDM compared to individuals without DM or GDM [34]. A study by Delgado et al. investigated the prevalence of GDM in PR and found it ranged from 3.2% to 4.5%, with an average prevalence of 3.68% and a trend toward increasing prevalence [35]. Within the US, Puerto Rican women had higher rates of GDM than other Hispanic subgroups, with 75.8 cases per 1000 live births [36]. In a study by Eick et al., approximately 40% of pregnant women in PR between 2005 and 2012 were classified as overweight or obese, which may predispose them to the development of DM and GDM [37]. However, these data are population-based and non-contemporaneous; therefore, causal inferences cannot be made. Although Puerto Rican women have an increased risk for DM and GDM, further research is warranted to determine whether a causal relationship exists between the higher rates of DM/GDM in PR and the observed differences in MMC incidence.

### 4.4. Folic Acid

When taken before and during pregnancy, folic acid has been shown to significantly reduce NTDs, such as MMC. It provides the one-carbon units required for DNA synthesis, repair, and methylation during early embryonic development, supporting neural tube closure and preventing MMC [38]. The U.S. Preventive Services Task Force recommends that women of childbearing age take 400 µg of folic acid per day, or 4 mg if they have previously had a pregnancy affected by NTD. This should begin at least one month before conception and continue throughout the first trimester, covering the neural tube closure window.

Lack of access to proper reproductive healthcare and inadequate sexual education in PR may hinder these prevention efforts. A study assessing folic acid consumption among women of reproductive age in PR found that only 30% of participants reported using folic acid supplements, with just 14% consuming it the day before the survey [39]. In contrast, a survey of women of reproductive age in California found that 40–41.1% of women took supplements containing folic acid [40]. These findings suggest that, despite a public health campaign initiated in 1994 to promote the use of folic acid in reproductive-aged women, folic acid supplementation remained inferior in PR.

Additionally, the use of folic acid supplementation among pregnant women is relatively high in the mainland US. Data from the National Health and Nutrition Examination Survey (NHANES) from 1999 to 2014 shows that approximately 77% of pregnant women reported using dietary supplements, with 64% specifically using prenatal supplements containing folic acid [41]. At the time, no such data is available for the Puerto Rican population. This discrepancy highlights the need for continued research and public health efforts to increase folic acid supplement use among women of reproductive age in PR to reduce the incidence of NTDs.

### 4.5. Sexual Education

Sexual education is also an essential factor to consider in the development of NTDs. Comprehensive sex education is an inclusive approach to teaching sexual and reproductive health that has been linked to lower rates of sexual activity among youth and increased use of contraception, which in turn decreases unintended pregnancies [42]. The United States Preventive Services Task Force (USPSTF) emphasizes that the risk of NTDs is higher in the absence of periconceptional folic acid supplementation, which has been proven to be less likely in unintended pregnancies [32]. Even though comprehensive sex education may increase awareness of reproductive health and the importance of preconception care, there is no evidence in the medical literature that it independently reduces the incidence of NTDs. However, a gap in adequate sexual education may exist in both PR and the continental US, as comprehensive sexual education has proven more effective than abstinence-only approaches in reducing unintended pregnancies and promoting healthier pregnancy planning [43].

This may be contributing to high rates of unintended pregnancies and, potentially, a higher incidence of NTDs. Further understanding of the specific curricula offered across different sectors of the continental US and PR, and their direct effects on NTD prevalence, is needed to reach conclusions on the impact of sex education on NTD prevalence.

### 4.6. Limitations

This study has several limitations. First, the analysis is based on aggregated population-level data, which restricts our ability to evaluate individual-level risk factors or establish causal relationships. Second, the relatively low annual numbers of births and myelomeningocele cases in PR may contribute to greater year-to-year variability in incidence rates, potentially magnifying fluctuations compared to the mainland US. Because this study relied on aggregated surveillance data, it was not possible to adjust for potential confounding variables such as maternal age, obesity, diabetes, socioeconomic status, or prenatal care utilization. These results should be interpreted as descriptive and hypothesis-generating rather than causal. Finally, there is limited availability of detailed clinical and behavioral data from pregnant women in PR, particularly regarding preconception health status, nutritional intake, and lifestyle habits during pregnancy. These gaps hinder more granular analyses that could clarify the contribution of modifiable maternal factors to the observed trends. Future studies incorporating individual-level data and expanded surveillance measures are warranted to better control for confounders and characterize the determinants of NTDs in this population.

## 5. Conclusions

This study reveals significant, persistent disparities in MMC incidence between PR and the US over the 25-year period of 1996–2020. While the US demonstrated a steady decline in MMC incidence, PR demonstrated consistently higher incidence rates and pronounced year-to-year variability. These differences remained even when analyzing the subset of data following folic acid fortification policies on the island (1999–2020). These findings underscore the need for organized, sustained public health efforts in PR to minimize MMC risk by improving preconception health, increasing folic acid intake, addressing metabolic and behavioral risk factors, and reshaping reproductive health education on the island. Although the underlying cause of this disparity is likely multifactorial, limited improvement suggests gaps in the implementation of public health initiatives and limited access to effective preventative medical care and health education likely play a significant role.

Our discussion explored potential contributing factors, including maternal metabolic comorbidities, genetic predispositions (e.g., MTHFR C677T homozygosity), lower folic acid supplementation, and deficiencies in reproductive health education. Although causality cannot be determined from surveillance data alone, the consistent disparity suggests that existing prevention efforts in PR may be insufficient, inconsistently applied, or inadequately targeted. Enhanced surveillance and further research into region-specific risk profiles are critical to reducing the preventable burden of MMC and achieving equity in congenital anomaly prevention across US jurisdictions.

## Data Availability

The data utilized in this study are publicly available. Data pertaining to the USA were extracted from the *National Vital Statistics Reports* published by the Centers for Disease Control and Prevention (CDC), National Center for Health Statistics (NCHS). Specifically, annual supplemental tables related to congenital anomalies by maternal age were retrieved from the CDC’s Vital Statistics online database for the years 1996 through 2020. Each report is accessible via the CDC’s National Vital Statistics Reports repository at https://www.cdc.gov/nchs/products/nvsr.htm (accessed on 7 July 2025) and through individual report links provided in the references. Data for PR were extracted from the Puerto Rico Birth Defects Surveillance and Prevention System (https://estadisticas.pr/files/Inventario/publicaciones/defectos%20congenitos%202016-2020.pdf) (accessed on 25 July 2025). No proprietary or confidential datasets were used. All data are freely available and can be accessed without restriction.

## Abbreviations

The following abbreviations are used in this manuscript:

MMC	Myelomeningocele
PR	Puerto Rico
US	United States
SD	Standard Deviation
CI	Confidence Interval
NTD	Neural Tube Defect
APC	Annual Percent Change
MTHFR	5,10-Methyltetrahydrofolate Reductase
PRASM	USA Pregnancy Risk Assessment Monitoring System
BRFSS	Behavioral Risk Factor Surveillance Systems
DM	Diabetes Mellitus
GDM	Gestational Diabetes Mellitus
NHANES	National Health and Nutrition Examination Survey
USPSTF	United States Preventive Services Taskforce
